# Next-generation sequencing of common osteogenesis imperfecta-related genes in clinical practice

**DOI:** 10.1038/srep28417

**Published:** 2016-06-23

**Authors:** Kristóf Árvai, Péter Horváth, Bernadett Balla, Bálint Tobiás, Karina Kató, Gyöngyi Kirschner, Valéria Klujber, Péter Lakatos, János P. Kósa

**Affiliations:** 1 1st Department of Internal Medicine, Semmelweis University, H-1083 Budapest, Korányi S. u. 2/a, Hungary; 2 PentaCore Laboratory, H-1094 Budapest, Bokréta u. 5, Hungary

## Abstract

Next generation sequencing (NGS) is a rapidly developing area in genetics. Utilizing this technology in the management of disorders with complex genetic background and not recurrent mutation hot spots can be extremely useful. In this study, we applied NGS, namely semiconductor sequencing to determine the most significant osteogenesis imperfecta-related genetic variants in the clinical practice. We selected genes coding collagen type I alpha-1 and-2 (*COL1A1, COL1A2*) which are responsible for more than 90% of all cases. *CRTAP* and *LEPRE1/P3H1* genes involved in the background of the recessive forms with relatively high frequency (type VII and VIII) represent less than 10% of the disease. In our six patients (1–41 years), we identified 23 different variants. We found a total of 14 single nucleotide variants (SNV) in *COL1A1* and *COL1A2*, 5 in *CRTAP* and 4 in *LEPRE1*. Two novel and two already well-established pathogenic SNVs have been identified. Among the newly recognized mutations, one results in an amino acid change and one of them is a stop codon. We have shown that a new full-scale cost-effective NGS method can be developed and utilized to supplement diagnostic process of osteogenesis imperfecta with molecular genetic data in clinical practice.

With the appearance of next generation sequencing (NGS) machines, molecular biology has entered in a new revolutionary phase. These new techniques combine high performance with much less expensive operation costs^1^. Thanks to the new benchtop sequencers, every laboratory has the opportunity to utilize next generation sequencing either as a research or a diagnostic tool. There is a variety of different methods in next generation sequencing. There is pyrosequencing, fluorescent detection of dNTP incorporation and last but not least semi-conductor sequencing technology^2^. Former sequencing systems used irreversible dye terminators of nucleic acid sequencing, as described by Sanger *et al*. all four terminators having different dyes^3^. This is the “gold standard” method, however, it is not only expensive but also a very time-consuming procedure. Next generation sequencing creates signals during the synthesis without the irreversible termination of the DNA strand, and all strands run in multiple wells at the same time. Therefore, the sequencing is massively paralleled, it could mean millions of DNA strands sequenced at once, thus giving much more information in much less time.

Our aim was to work out a NGS method utilizing the IonTorrent PGM (ThermoFisher Scientific, Waltham, MA, USA) in diagnostic settings for osteogenesis imperfecta (OI). Osteogenesis imperfecta or brittle bone disease is a rare genetic disorder - with dominant or recessive inheritance pattern-of the skeletal system. The background of the disease in most cases is a mutation of the genes encoding collagen type I molecule complex^4^. In a smaller, but significant number of cases the disease is caused by variants in other genes related to collagen synthesis^5^,^6^. Type I collagen is a commonly found protein in the body: it is in the walls of the viscera and the vessels, in the skin and in the bone tissue^7^. Bone has two parts: the organic part, which is the matrix synthesized by bone cells or osteoblasts, and the inorganic part, which means calcium salts deposited in the matrix^8^. In osteogenesis imperfecta, the corrupted collagen cannot form the appropriate matrix which leads to a brittle, fragile skeletal system^9^.

Type I collagen has a helical structure. It is built up by two strands of alpha-1 protein and one strand of alpha-2 protein. Alpha-1 is encoded by *COL1A1*, alpha-2 is encoded by *COL1A2*^10^. These genes have a relatively high number of exons, which makes them complicated to sequence by conventional methods. Both *COL1A1* and *COL1A2* contain 52 exons^11^. In addition, no dramatic mutation ‘hot spots’ have been identified within these genes. Because of this, almost 2,500 different *COL1A1* and *COL1A2* mutations have been reported and listed in the Osteogenesis Imperfecta Variant Database (http://www.le.ac.uk/ge/collagen/).

First, this disease was believed to be caused by the mutation of these two genes and OI had four categories (Sillence Classificaton), however, it turned out that Type IV has several different subtypes. Among these subtypes, there are cases where OI is caused by the variations of the proteins that facilitate the appropriate folding of type I collagen^11^,^12^,^13^,^14^,^15^,^16^. Microscopic studies of OI bone identified people who are clinically within Type IV group but have distinctive patterns to their bone. As a result of this research, Type V and VI were added to the Sillence Classification. Two recessive types of OI, Types VII and VIII, were identified in 2006. Unlike the dominantly inherited types, the recessive types of OI do not involve mutations in the type I collagen genes. These recessive types of OI result from mutations in the cartilage-associated protein gene (*CRTAP*) and the prolyl 3-hydroxylase 1 gene (*LEPRE1/P3H1*) genes^17^,^18^. In the past years, recessive OI types due to very rare defects in additional genes (e.g. *PEDF, HSP47, FKBP65 and BMP-1*) have been described in the literature^6^,^19^. Different types of OI could have similar clinical features, thus, the definitive diagnosis can be revealed by analyses of the genes lying in the background of the disease.

## Results

### Descriptive results from sequencing runs

After chip loading, the Ion Sphere Particles (ISPs) density was between 64–87% (average: 77.66%). Average number of total reads was 2.842.181 (range: 1.714.250–3.506.477). Average number of reads on target was 436.086. Average target coverage 1x was 98.65% with a raw mean accuracy of 99.03%. The average base coverage depth was 779 (118–1769). In the six patients, we identified 23 different variations (Table 1). We found 7 in *COL1A1* and 7 in *COL1A2*.

We have identified three novel variations. Two of them, namely c.189C > A (*COL1A1*) and c.811G > T (*COL1A2*) are considered as pathogenic. Variant c.189C > A is located in exon 2 of the *COL1A1* gene. The variant causes premature termination of translation and nonsense mediated decay of the mRNA as it is a stop codon mutation. The c.811 G > T: p.Gly271Cys is positioned in *COL1A2*. Position c.811 in exon 17 was previously recognized as a locus for pathogenic variant (G > C substitution, causing a glycine > arginine amino acid change), however, no G > T substitution was ever described. This G > T transversion results in a glycin > cysteine change at amino acid level. Prediction softwares PolyPhen-2 and SIFT classify both alteration in the same deleterious or damaging category. In case of both G > C and G > T nucleotide substitution, the uncharged, apolar glycine is switched to charged amino acids with polar side chains. The targeted checking of c.811G > T variant was carried out and we detected it in the affected family members showing OI phenotype.

We have found a variant, namely c.2072G > A: p.Gly691Asp which is located in the coding sequence of exon 31 of *COL1A2*. According to the applied prediction softwares, this variant damages the protein structure. The presented G > A transition results in a glycine to aspartic acid alteration. Collection of segregation data in the presence of this variant was not available, thus, we considered it as a variant with uncertain significance (VUS).

Furthermore, we have also described two already established pathogenic variants, c.391C > T (*COL1A1*) and c.750+1G > A (*COL1A1*). The c.391C > T substitution causes a stop codon (p.Arg131Ter) in exon 5. The other mutation, c.750+1G > A is a splice site variant in intron 10.

All of the called variants with clinical significance were validated by Sanger sequencing. No false positive samples were seen in the Ion Torrent reads.

### Results of the individual patients

We enrolled 6 patients in our study, four males and two females. Age range was between 1–41 years. Bone mineral density (BMD) was measured at the lumbar spine in participants and decreased BMD were detected compared to the normal age-matched values. Detailed patients’ information can be found in Table 2.

## Discussion

We were among the first who apply NGS technology to analyze osteogenesis imperfecta-related genes in clinical settings. In this study, we attempted to demonstrate the huge potential of NGS in the clinical practice of OI affected patients. In most of the cases, genetic results support the accurate diagnosis as well as significantly contribute to the subclassification of OI, especially in mild phenotypes.

Osteogenesis Imperfecta Variant Database contains genetic variants from 19 genes (http://www.le.ac.uk/ge/collagen/). We decided to target four of them (*COL1A1, COL1A2, CRTAP, LEPRE1/P3H1*) because genes coding collagen type I alpha-1 and -2 chains are responsible for more than 90% of all cases^4^,^20^. Genes involved in the background of the recessive forms with relatively high frequency (type VII and VIII) represent less than 10% of the disease^19^.

Genes related to OI usually contain numerous coding exons (total 126 exons of 4 genes in our study). The large number of the targeted genomic regions and the lack of mutation hot spots, resulting in a very time- and cost-demanding molecular analysis when diagnosis is set up by classic Sanger sequencing. Wang *et al*. demonstrated the efficacy of high resolution melting (HRM) analysis of *COL1A1* and *COL1A2* genes in OI subjects^21^. This is a pre-screening technology, therefore, every identified genetic variant needs to be confirmed by Sanger sequencing. In contrast, using NGS, the verification is necessary only for the pathogenic mutations. Sule *et al*. also presented the accuracy of NGS platform in patients with various disorders of low and high bone mineral density in a clinical setting^22^.

We have developed our NGS method utilizing IonTorrent PGM from ThermoFisher Scientific. This benchtop sequencer belongs to the semiconductor sequencer family. It acquires the DNA sequence by detecting electric impulses created by the release of H^+^-ions in its microchip. The solution which contains the H^+^-ions serves as a gate electrode of a transistor, a so-called ion-sensitive field electricity transistor (ISFET)^23^. Using this technique, we have identified a total of 23 different variants in the selected 4 genes at the same time in a cost-effective manner.

When a genetic test panel is created for the clinical practice, it is important, that it contains only well-established genes with clear relationship to the disorder^24^. According to the latest guideline from the American College of Medical Genetics and Genomics (ACMG), it is necessary to limit the number of variants with uncertain significance (VUS) as low as possible^25^. The probability to find VUSs is getting higher as the targeted areas of the genome are increasing. VUSs can confuse both the clinicians and the patients and can put unnecessary burden to the patients shoulder. Moreover, such findings can lower the clinical utility of a genetic test. Using our approach, one can test the patients suspect for OI by the IonTorrent method to identify most of the pathogenic variants, and a more extended gene panel is required only when the common OI-associated genes are intact or the family anamnesis is unclear.

Osteogenesis imperfecta is a perfect example to show NGS potential to be used effectively. It is relatively easy to set up the diagnosis of OI but it is really difficult to inevitably identify the causative genetic alteration in each patient. Researchers find more and more genes and variations playing role in the development of the disease. In our study, we recognized 1 novel variant in the *COL1A1* gene and 2 new alterations in *COL1A2* gene which are not present in current databases.

Mutation c.189C > A in *COL1A1* gene causes premature termination of collagen type I alpha-1 chain synthesis. Position c.811 in *COL1A2* exon 17 was previously identified as a locus for pathogenic variants, resulting in a glycine > arginine change. Our newly determined G > T substitution modified glycine to cysteine at amino acid level. This novel alteration is categorized as deleterious. Partly, because cysteine can form disulfide bridges which may have a significant impact on the COL1A2 protein conformation by prediction softwares. Moreover, the clinical relevance of c.811G > T variation was strengthened by segregation data from family screening. All of the affected family member (mother and younger sister) carry this mutation and also show clinical symptoms of OI.

Position c.2072A > G is located in the 31^st^ coding exon of *COL1A2* which is a glycine > aspartic acid change at protein level, replacing an apolar amino acid to a polar, negatively charged type. According to mutation effect prediction algorithms, this variant damages the structure of alpha-2 chain. There is no family history of OI in case of this patient, and this variant might be a *de novo* mutation, however, family screening was not possible. Thus, we defined c.2072A > G as a VUS.

Two rare missense variants, namely c.4313C > G in *COL1A1* gene and c.655G > A in *CRTAP* gene were found in our cohort. Even though both alterations cause amino acid changes, there is no exact literature data about their pathogenicity. Moreover, in each cases they co-occure with a well-defined disease causing mutation (in patient 4 and in patient 6) Based on this information, we suggest that the identified two rare missense variants should be characterized as likely benign.

The exact diagnosis is relevant since it might have an impact on the treatment of the individual, and it is also important when it comes to family planning and genetic counseling. In many cases, two healthy parents has an OI affected child and it could be mandatory to know if the child has a new *de novo* mutation or it has the recessive inheritance of the disease^26^. To decide between these questions and to avoid the unnecessary invasive prenatal test during the next pregnancy, genetic analysis of the proband utilizing NGS technology can give rapid, comprehensive and accurate answer.

In summary, we have shown that a new full-scale cost-effective NGS method can be developed and utilized to supplement diagnostic process of osteogenesis imperfecta with molecular genetic data in clinical practice.

## Materials and Methods

### Biological samples and DNA isolation

Six Caucasian patient samples were selected and anonymized for this study. Five patients were unrelated and patient 1 was related to patient 2. DNA was isolated from 200 μl of peripheral blood using Reliaprep Blood gDNA Miniprep System according to the manufacturer’s instructions (Promega, Fitchburg, WI, USA). The concentration of the isolated DNA was determined by Qubit dsDNA HS Assay Kit (ThermoFisher Scientific, Waltham, MA, USA). The study was approved by the Semmelweis University’s Committee of Research Ethics, and all patients gave written informed consent. All experiments were performed in accordance with relevant guideline and regulation.

### Capture design

The target list was carefully prepared based on literature data and the information in NIH Genetic Home Reference site (http://ghr.nlm.nih.gov/). We selected four genes for the analysis. Mutations in *COL1A1* (4395 bp, 52 exons) and *COL1A2* (4101 bp, 52 exons) genes are responsible for approximately 90% of all OI cases. Mutations in the *CRTAP* (1206 bp, 7 exons) and *LEPRE1* (2892 bp, 15 exons) genes are in the background of a rare milder form of osteogenesis imperfecta (type VII, VIII). SureDesign software (Agilent, Waldbronn, Germany) was used to design the custom HaloPlex capture assay. We selected all of the coding exons of the following genes from RefSeq database and added an extra 10 bases upstream from 3′ end and extra 10 bases downstream from 5′ end: *COL1A1* (100%), *COL1A2* (99.05%), *CRTAP* (100%) and *LEPRE1* (100%) with 99.76% of total coverage. In case of *COL1A2*, the coverage is 99.05%, because the assay software was not able to design capture probes for codons between 234 to 246. The detailed coverage of the targeted genes is shown in Supplementary Fig. 1. Total amplicon number was 2036, target size 15.25 kb and the total design was 45.63 kb.

### Sequence capture and library preparation

For sequence capture, HaloPlex Target Enrichment System Kit, ION (Agilent, Waldbronn, Germany) was used, according to the manufacturer’s instructions. Briefly, in the first step, 225 ng (5 ng/μl) gDNA samples are digested in eight different restriction reactions, each containing two restriction enzymes, to create a library of gDNA restriction fragments. The digestion lasted 30 minutes at 37 °C. In the second step, the collection of gDNA restriction fragments is hybridized to the HaloPlex probe capture library. HaloPlex probes are designed to hybridize selectively to fragments originating from target regions of the genome and to direct circularization of the targeted DNA fragments. During the hybridization process, Ion Torrent sequencing motifs, including IonXpress barcode sequences, are incorporated into the targeted fragments. The hybridization process lasted 3 hours at 54 °C after 10 minutes of denaturing step at 95 °C. In the third, capture step, the circularized target DNA-HaloPlex probe hybrids, containing biotin, are captured on streptavidin beads. HaloPlex Magnetic Beads were added to the DNA-HaloPlex probe hybrids, supernatants were removed and Capture Solution was added to the magnetic beads. After 15 minutes of incubation at room temperature, the Capture Solution was removed from the beads and Wash Solution was added to the samples and they were incubated for 10 minutes at 46 °C, then Wash Solution was discarded. In the next step, DNA ligase is added to the capture reaction to close nicks in the circularized HaloPlex probe-target DNA hybrids. The sample tubes were incubated in a thermal cycler at 55 °C for 10 minutes, using a heated lid. When the 10-minute ligation reaction period is complete, the following step is to elute the captured DNA libraries with 50 mM NaOH solution. The final step of the library preparation is the PCR amplification of the captured target libraries with the following mixture per sample: 10 μl 5X Herculase II Reaction Buffer, 0,4 μl dNTPs (100 mM), 1 μl HaloPlex ION Primer 1 (25 μM), 1 μl HaloPlex ION Primer 2 (25 μM), 0,5 μl 2 M Acetic acid, 1 μl Herculase II Fusion DNA Polymerase, 16,1 μl Nuclease-free water followed by 2 minutes incubation at 98 °C, than 21 cycles of 98 °C 30 sec, 60 °C 30 sec and 72 °C 1 minute with a final 10 minutes of elongation at 72 °C. The amplified target DNA is purified using AMPure XP beads (Beckman Coulter). The concentration of the captured libraries was determined by Qubit dsDNA HS Assay Kit (ThermoFisher Scientific, Waltham, MA, USA).

### Ion Torrent sequencing

The HaloPlex libraries were diluted to 26 pM concentration, then 20 μl of diluted library was added into the emulsion PCR with ISPs using automated template preparation on Ion One Touch (ThermoFisher Scientific) instrument with Ion One Touch v2 DL kit (ThermoFisher Scientific). As a result of this reaction, amplicons were clonally amplified and bound to the surface of the ISPs. Non-templated beads were removed from the solution in an automated enrichment step using Ion One Touch ES instrument (ThermoFisher Scientific). ISPs were loaded into Ion 316 chips and the sequencing runs were performed using Ion PGM 200 Sequencing kit (ThermoFisher Scientific) with 500 flows.

### Validation Sanger sequencing

The PCR primers were designed using Primer3Plus (http://primer3plus.com/) software. Roche FastStart TaqMan Probe Master (Roche, Basel, Switzerland) kit was used to amplify the target regions and the PCR program was as follows: 95 °C 10 minutes, 40 cycles of 95 °C 30 sec, 60 °C 30 sec, 72 °C 45 sec and the final step was 72 °C 5 minutes. PCR products were enzymatically cleaned using ExoSAP IT (Affymetrix, Santa Clara, CA, USA) according to the manufacturer. Sanger sequencing was performed using BigDye Terminator v3.1 Cycle Sequencing Kit (ThermoFisher Scientific) using an ABI 3130 instrument (ThermoFisher Scientific).

### Data analysis

Data from the Ion Torrent runs were analyzed using the platform-specific pipeline software Torrent Suite v3.2.1 to base-calling, trim adapter and primer sequences, filter out poor quality reads, and de-multiplex the reads according to the barcode sequences. Briefly, TMAP algorithm was used to align the reads to the reference genome (hg19) and then the variantCaller plugin was selected to run to search for germline variants in the targeted regions. Variants were reviewed and annotated using dbSNP (http://www.ncbi.nlm.nih.gov/projects/SNP/) and the Osteogenesis Imperfecta Variant Database (oi.gene.le.ac.uk). For variant interpretation Ingenuity Variant Analysis Pipeline (Ingenuity Systems Inc., Redwood City, CA, USA) was also used. Called and deleterious variants were Sanger sequenced for validation. The Sanger sequences data were investigated using ABI Sequence Scanner 1.0 (ThermoFisher Scientific).

Variant classification is based on the current ACMG standards and guidelines 2015^27^.

## Additional Information

**How to cite this article**: Árvai, K. *et al*. Next-generation sequencing of common osteogenesis imperfecta-related genes in clinical practice. *Sci. Rep.* **6**, 28417; doi: 10.1038/srep28417 (2016).

## Supplementary Material

Supplementary Information

## Figures and Tables

**Table 1:** List of variants in six patients sorted by genes.

Gene ID	dbSNP	Coding variant	Protein effect	Variant type	MAF
***COL1A1***	**N/A**	**c.189C > A**b>†	**p.Cys63Ter**	**pathogenic**	**N/A**
***COL1A1***	**N/A**	**c.391C > T**	**p.Arg131Ter**	**pathogenic**	**N/A**
***COL1A1***	**N/A**	**c.750** **+ 1G >1 A**	**Splice site**	**pathogenic**	**N/A**
*COL1A1*	N/A	c.2427C > G	p.(=).Gly809	synonym variant	N/A
*COL1A1*	rs17857117	c.4313C > G	p.Pro1438Arg	missense, non-pathogenic	N/A
*COL1A1*	rs1800215	c.3223A > G	p.(=).Ala1075	synonym variant	T = 0.0212/106
*COL1A1*	rs2734272	c.2298T > C	p.(=).Thr766	synonym variant	A = 0.0002/1
***COL1A2***	**N/A**	**c.2072G > A**†	**p.Gly691Asp**	**VUS**	**N/A**
***COL1A2***	**N/A**	**c.811G > T**†	**p.Gly271Cys**	**pathogenic**	**N/A**
*COL1A2*	rs1800222	c.246T > C	p.(=).Asp82	synonym variant	C = 0.312/682
*COL1A2*	rs1800248	c.3135C > T	p.(=).Gly1045	synonym variant	T = 0.089/195
*COL1A2*	rs42519	c.937-3C > T	Splice site	intronic	C = 0.145/317
*COL1A2*	rs42524	c.1645C > G	p.Pro549Ala	missense, non-pathogenic	C = 0.147/321
*COL1A2*	rs412777	c.1446A > C	p.(=)Pro482	synonym variant	C = 0.3273/1639
*CRTAP*	rs1135127	c.1032T > G	p.(=).Thr344	synonym variant	G = 0.281/614
*CRTAP*	rs1135128	c.1044G > A	p.(=).Ser348	synonym variant	A = 0.281/614
*CRTAP*	rs11558338	c.213G > A	p.(=).Leu71	synonym variant	A = 0.260/568
*CRTAP*	rs4076086	c.534C > T	p.(=).Asp178	synonym variant	T = 0.300/656
*CRTAP*	rs145048208	c.655G > A	p.Gly219Ser	missense variant	N/A
*LEPRE1*	rs3738496	c.2129T > A	p.Phe710Tyr	common missense variant	T = 0.141/308
*LEPRE1*	rs4660662	c.2148T > C	p.(=).Gly716	synonym variant	N/A
*LEPRE1*	rs116577636	c.2248G > A	p.Gly750Arg	missense variant	T = 0.0012/6
*LEPRE1*	rs11581921	c.1647G > A	p.Met549Ile	common missense variant	T = 0.0575/288

It is clearly visible that the most polymorphic genes are *COL1A1* and *COL1A2*. The most of the pathogenic variants were found in *COL1A1*. The † symbol marks the newly identified variants. Position c.811G > T is a known locus for pathogenic variants, however no guanine > thymine change was previously described. MAF: minor allele frequency from dbSNP database, N/A: not available.

**Table 2:** Patient characteristics and genetic variants with clinical significance in OI.

	Gender	Age (yrs)	Fractures*	BMD (L2–L4) g/cm^2^	Variants with clinical significance	Family history	Blue sclera	Hearing loss
Patient 1†	Male	9	3	0.381	c.391C > T (*COL1A1*)	Father and paternal grandmother has OI	Yes	N/A
Patient 2	Male	41	10	N/A	c.391C > T (*COL1A1*)	Mother has OI	No	Yes
Patient 3	Male	18	3	0.685	c.2072G > A (*COL1A2*)‡	No	No	Yes
Patient 4	Female	2	3	0.390	c.189C > A (*COL1A1*)	Mother has blue sclera, but no fractures	Yes	N/A
Patient 5	Female	1	3	0.170	c.811G > T (*COL1A2*)	Mother had 7 fractures	No	No
Patient 6	Male	1	1	0.167	c.750 + 1G > A(*COL1A1*)	No	Yes	N/A

Bone mineral density (BMD) was measured at the lumbar spine by dual-energy X-ray absorptiometry (DPX-L, Lunar Corp. Madison, WI, USA).

^*^History of recurrent low-energy fractures: Patient 1 had 2 fracture events with 3 fragility fractures (tibia, fibula and radius). Patient 2 had approximately 10 fractures. Patient 3 had 3 fracture events with 3 fragility fractures (skull, clavicular and femoral neck). Patient 4 had 3 fracture events with 3 fragility fractures (tibia, femur and humerus). Patient 5 had 3 fracture events with 3 fragility fractures (tibia, femur). Patient 6 had 1 fracture event with a femoral fracture.

^†^Patient 1 is the son of Patient 2.

^‡^Variant c.2072G > A (*COL1A2*) classified as VUS.
