# Supplementary material for: Next-generation sequencing of common osteogenesis imperfecta-related genes in clinical practice
Source: Sci Rep. 2016 Jun 23;6:28417. doi: 10.1038/srep28417 (PMC4917842; doi:10.1038/srep28417)
Supplement: Supplementary Information [file srep28417-s1.pdf]

Next-generation sequencing of common osteogenesis imperfecta-related genes in clinical practice

KRISTÓF ÁRVAI, PÉTER HORVÁTH, BERNADETT BALLA, BÁLINT TOBIÁS, KARINA KATÓ, GYÖNGYI KIRSCHNER, VALÉRIA KLUJBER, PÉTER LAKATOS, JÁNOS P. KÓSA

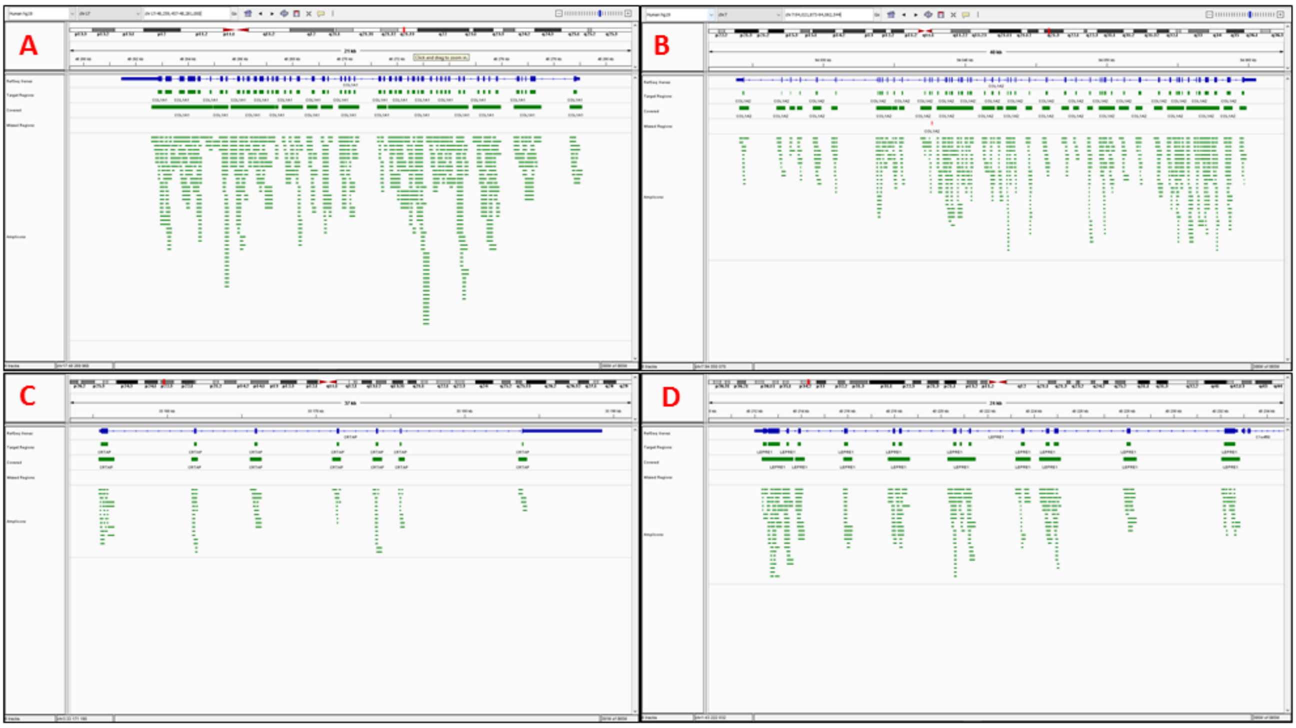

Supplementary figure 1.

The full targeted genes in Integrative Genomics Viewer (IGV) browser are shown. The track named 'covered' indicating those genomic regions which have been analyzed in our four genes. Amplicon track shows the Haloplex DNA fragments which have been actually sequenced. A: COL1A1 B: COL1A2 C: CRTAP D: LEPRE1
